# Effects of a Massage Protocol in Tensiomyographic and Myotonometric Proprieties

**DOI:** 10.3390/ijerph18083891

**Published:** 2021-04-08

**Authors:** Albert Pérez-Bellmunt, Noé Labata-Lezaun, Luis Llurda-Almuzara, Jacobo Rodríguez-Sanz, Vanessa González-Rueda, Elena Bueno-Gracia, Derya Celik, Carlos López-de-Celis

**Affiliations:** 1 Faculty of Medicine and Health Sciences, Universitat Internacional de Catalunya, c/Josep Trueta s/n., 08195 Barcelona, Spain; aperez@uic.es (A.P.-B.); nlabata@uic.es (N.L.-L.); lllurda@uic.es (L.L.-A.); jrodriguezs@uic.es (J.R.-S.); vgonzalez@uic.es (V.G.-R.); 2 ACTIUM Research Group, 08195 Barcelona, Spain; 3 Faculty of Health Sciences, Unviersity of Zaragoza, 50009 Zaragoza, Spain; ebueno@unizar.es; 4 Department of Physiotherapy and Rehabilitation, Faculty of Health Science, Istambul Univeristy-Cerraphpasa, Istanbul 34147, Turkey; derya.celik@istanbul.edu.tr

**Keywords:** massage, musculoskeletal manipulations, sport, physical therapy, athletic performance, muscle tonus

## Abstract

Background: Pre-competition massage is usually used to improve athletic performance and reduce risk of injury. Despite its usual use, the effects of pre-competition massage on neuromuscular function have barely been studied. The aim of this study is to evaluate the effects of the pre-competition massage over the gastrocnemius neuromuscular function. Method: The study is a quasi-experimental clinical trial thirty healthy athletes were enrolled in the study. Subjects received an intervention in one leg (experimental), consisting of a massage, and no intervention in the opposite leg (control). From all values of neuromuscular function, the following were analyzed: contraction time (Tc) and maximal displacement (Dm) by tensiomyography, and stiffness and tone by myotonometry. Results: Main effects of pre-competition massage on neuromuscular function include a significant (*p* < 0.05) increase in Tc and Dm variables, as well as a reduction in stiffness and tone. Conclusion: Data shows an increase in Tc and maximal radial displacement (Dm) variables, as well as a reduction in stiffness and tone. More quality studies are needed to draw clear conclusions about the effects of pre-competition massage.

## 1. Introduction

Massage is one of the most used tools not only in the field of rehabilitation but also in sports [1,2]. From this technique, pre-competition massage has emerged, with the main objective of improving the performance of athletes and reducing the risk of injury [3]. Among the main physiological mechanisms of pre-competition massage is local hyperemia, which leads to an increase in the supply of oxygen to the tissues on which it is applied [4,5,6], in order to simulate an active warm-up [5]. Some studies have shown that massage techniques can produce an improvement in grip strength [7], range of motion (ROM) [8,9] and delayed onset muscular soreness [10]. However, there is controversy as to whether pre-competitive massage produces improvements in specific athletic performance parameters [11]. Pre-competition massage is used in many sports competitions. On a physiological level, massage techniques have been shown to increase blood perfusion and cell proliferation [12,13], which may have a positive effect on tissue in some sports disciplines such as long-distance running. A recent systematic review found that massage increases skin microcirculatory flow motion not only locally but also beyond, affecting systemic hemodynamics. This observation is an interesting example of the efficacy of cardiovascular integration mechanisms involving distal microcirculatory homeostasis [14]. On the other hand, it is not known whether pre-competition massage can generate a change in muscle tone and activity that may be detrimental to other sports disciplines involving faster movements such as sprints [15].

In recent decades, two novel instruments that allow the assessment of both neuromuscular function (NMF) and the mechanical and contractile properties of soft tissues have been introduced [16]. These techniques are tensiomyography (TMG) and myotonometry (MMT) [17]. Their main advantages include that they are portable, non-invasive, inexpensive, and take little effort on the part of the athlete [18,19,20,21,22,23].

Several techniques have tried to modify the NMF with the purpose of improving athletic performance [11]. Nonetheless, studies evaluating the effects of a massage in NMF have not been found. For these reasons, the aim of this study is to evaluate the acute effects of a pre-competition massage in gastrocnemius neuromuscular function [24,25,26].

## 2. Materials and Methods

### 2.1. Study Design

A quasi-experimental clinical trial was carried out. Participants received intervention in one leg (experimental limb) consisting in a massage, and no intervention in the opposite leg (control leg). The tested leg was randomized using <www.random.org> website, accessed on 3 March 2021. The assessor was blinded about the treated leg.

### 2.2. Participants

Subjects were healthy athletes studying in the Faculty of Medicine and Health Sciences of the Universitat Internacional de Catalunya, who participated voluntarily after signing an informed consent. The protocol of the study was registered in <www.clinicaltrials.gov> under the reference code NCT03941067. The study protocol was approved by the local ethics committee (CER-UIC-Barcelona; study code: CBAS-2018-29), and it was carried out respecting data protection law, according to the Helsinki declaration [27].

A pilot study (n = 10) was carried out in order to set the sample calculation [28]. Accepting an alpha risk of 0.05 and a beta risk of 0.2 in a bilateral contrast, 30 subjects in the first group and 30 in the second group were required to detect a difference equal to or greater than 5.67 ms in the contraction time (Tc) variable. The common standard deviation was assumed to be 7.22. A follow-up loss rate of 15% has been estimated.

Inclusion criteria were: (a) age between 18 and 40 years; (b) healthy athletes, with no lower limb pathology; and (c) to have signed the informed consent. Exclusion criteria included: (a) pregnant women; (b) subjects presenting neurologic or orthopedic problems during the last year; (c) having received any lower limb surgical interventions during the last 6 months; (d) not understanding the orders provided by the investigators; (e) metastasis or serious tumor processes; and (f) musculoskeletal alterations that did not allow the subject to perform the study protocol; (g) body mass index (BMI) of 40 kg/m^2^ or higher.

Subjects were instructed to come with the following conditions: not having performed strenuous exercise in the previous 48 h; not having any intake of energy drinks, caffeine, or alcohol in the previous 3 h; and not having intake of any food 2 h before performing the analysis [16].

### 2.3. Outcome Measures

Demographic and anthropometric variables were collected at the beginning of the study (Table 1). NMF was measured before and after the intervention. Measurements were taken in both treated and non-treated limbs. The neuromuscular function variables were measured in the medial head of gastrocnemius muscle, because in sports this head muscle presents a higher incidence of injury than the lateral head [29]. To make these measurements, subjects were placed lying prone over a stretcher and with a cushion under the ankles to get a small knee flexion. Subjects were asked to perform an isometric contraction of the gastrocnemius muscle, in order to obtain the maximum displacement point of the muscle belly, which would be used as a reference for pre and post-intervention measurements [16].

**Figure 1:**
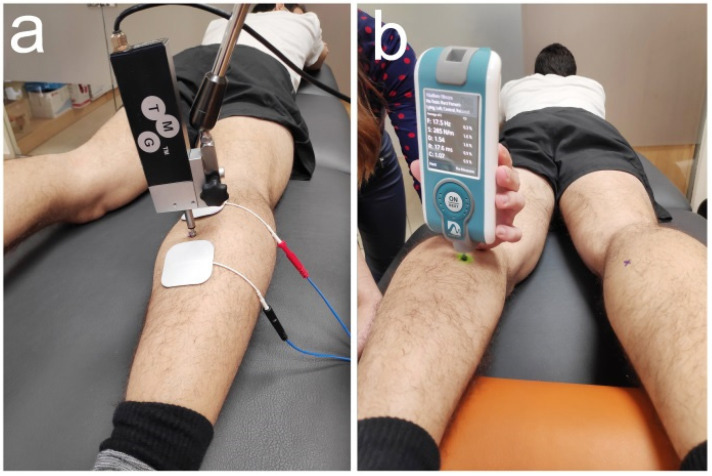
(**a**) Tensiomyography; (**b**) Myotonometry.

Some studies have demonstrated that TMG has a good intra-observer, intra-session, and between days reliability, for lower limbs muscles [30,31]. TMG evaluation was done using a Dc–Dc Trans-Tek^®^ transductor (GK 40, Panoptik d.o.o., Ljubljana, Slovenia) placed over the skin, on the maximum displacement point of the muscle belly contraction (Figure 1). Two self-adhesive electrodes (TMG electrodes, TMG-BMC d.o.o., Ljubljana, Slovenia) were placed equidistant, proximal (anode) and distal (cathode), to the sensor. Electrical stimulation was applied through a TMG-100 System electrostimulator (TMG-BMCd.o.o., Ljubljana, Slovenia) with intensities of 20, 40, 60, 80, and 100 mA, with a duration of 1 ms. Maximum displacement was chosen to get all the outcome measurements of the TMG [16]. TMG variables were the following: maximal displacement (Dm), which is the maximal radial displacement produced in the muscle belly after the electrical stimulation, measured in mm. The rest of the variables were calculated from Dm. These included delay time (Td), described as the time between the electrical stimulation and 10% of the Dm; contraction time (Tc), described as time between 10% and 90% of Dm; sustain time (Ts) of the contraction, going from 50% of maximum contraction to 50% of maximum relaxation; and relaxation time (Tr), which is the time between 90% of maximum contraction and 50% of maximum relaxation.

Myotonometry is a valid and reliable device that allows assessment of the viscoelastic properties of the muscle in a non-invasive way [32]. Moreover, the MyotonPRO shows good intra-operator repeatability in gastrocnemious muscle [33]. Myotonometry was measured with a MyotonPro (MytonPro, Myoton Ltd.s., Tallinn, Estonia). During measurements, MyotonPro was placed over the surface of the skin, perpendicular to the tissue, and located over the maximum displacement point of the muscle belly. From this position, a small pressure established by the MyotonPro was made against the tissue, so that the device could make three short applications of pressure (15 ms) on the skin [22,34,35,36,37,38]. With this measurement, the following outcomes were calculated: tone, natural oscillation frequency (Hz); stiffness, as the resistance to a contraction or to an external force that deforms its initial shape (N/m); and relaxation, as time for a muscle to recover its shape after a voluntary contraction or removal of an external force (ms) [18,39].

### 2.4. Intervention

Treatment of the experimental leg consisted in a pre-competition massage on the posterior side of the leg, over the triceps surae muscles. Duration of the massage was 5 min, and it was applied with a neutral massage cream by a physical therapist with more than 5 years of experience. The sequence of the intervention was an adaptation of a previous used method [40]: 1 min of high frequency effleurage; 1 min of high frequency petrissage; 1 min of high frequency vibrations; 1 min of high frequency petrissage; and 1 min of high frequency effleurage. The control leg did not receive any type of therapy between measurements. After completion of the massage protocol, all massage cream was removed, and an independent investigator performed the measurements without knowing which was the experimental leg. Thus, despite not being able to blind the participants, blinding of the investigators was achieved.

### 2.5. Statistical Analysis

For statistical analysis, SPSS 21.0 software (IBM SPSS Statistics, Chicago, IL, USA) was used. Descriptive analysis was carried out. For quantitative variables, mean and standard deviation were calculated. Frequencies were calculated for demographic and anthropologic qualitative variables.

For the NMF variables, all of the quantitative and descriptive statistics were calculated. Normality assumption was assessed using the Shapiro–Wilk test, in order to know whether to use parametric or non-parametric tests.

Differences from baseline to post-intervention data were observed for experimental and control limbs. A paired *t*-test was used for normally distributed variables, and a Wilcoxon test was conducted for non-normally distributed variables.

Differences between groups were observed using an unpaired *t*-test for those variables normally distributed, and a Mann–Whitney U test for those with non-normal distribution. The effect size was calculated by Cohen’s d test. An effect size >0.8 was considered large; around 0.5, intermediate; and <0.2, small.

Significance level was set at *p* < 0.05.

## 3. Results

Out of 32 recruited subjects, two were excluded for not meeting the inclusion and exclusion criteria. A total of 30 volunteers completed the study: 10 were women (33.3%) and 20 men (66.7%), with a mean age of 23.87 years old (SD ± 6.17), a mean weight of 69.23 kg (SD ± 1.76), and a mean height of 1.74 m (SD ± 0.09) (Table 1).

The NMF outcomes related to the before and after comparison are reflected in Table 2.

The findings of the differences in the NMF between the experimental and control limbs are presented in Table 3.

## 4. Discussion

Massage is a tool used to improve athletic performance. The objective of this study was to evaluate changes in NMF after a pre-competition massage session. Main results show significant differences in Tc and tone and stiffness after the pre-competition massage. The Tc and Tone differences are statistically significant in comparison with the control leg.

Regarding to all TMG parameters, previous studies have shown that the most reliable ones are Dm and Tc [41,42]. Main results of the present study show that Tc increases significantly in the experimental limb. As Rusu et al. [43] mentioned, a decrease in contraction time (Tc) is related to a greater recruitment of type II fibers (fast twitch fibers). Moreover, according to Haff et al. [44], type II fibers are the main fibers related to the maximum force production and explosive movements, essentials in athletic performance, especially in high-speed sports such as soccer or sprinting. In fact, lower Tc values have been observed in power athletes compared to endurance athletes [23,45] and between power and endurance master athletes [46]. Therefore, results obtained in our study suggest that pre-competition massage could decrease the activation of this type of fibers, increasing the contraction time. Increases in Tc values have also been observed in muscle fatigue situations [47] or as a result of the application of cold water immersion [48], often related to post-exercise recovery measures [49].

Furthermore, Alentorn-Geli et al. [50] stated that an increase in Tc values of hamstring muscles could be related to an increase in the risk of ACL (anterior cruciate ligament) injury, due to the role of these muscles in the control of the anterior slide of the tibia. In addition, several studies have shown the role of the triceps surae in stabilizing the knee joint, working as an agonist of the ACL [51,52]. It could be assumed that an increase in contraction time of this muscle, as a result of the application of a massage protocol, may increase the ACL loads. Given these data, an increase in contraction time caused by the pre-competition massage could increase the injury risk in high-speed sports. Unlike massage, active protocols have been shown to be more effective in decreasing Tc values [53] and could be a better alternative.

Although not statistically significant, Dm values show an increase in the experimental limb. An increase in the Dm is related to less muscular stiffness [54]. A low degree of stiffness could be associated with reduced movement efficiency [55], although differences between power and endurance athletes have also been found, with higher Dm values in endurance athletes [45]. High Dm values of the hamstring and quadriceps muscles have also been found in soccer players who have suffered an ACL injury, compared with healthy soccer players. [50].

In relation to stiffness and tone measured by myotonometry, a statistically significant reduction can be observed in the experimental limb. These data agree with the results obtained in Dm variable, since all three variables are related to the degree of muscle deformation before a stimulus [16]. Moreover, similar results in all the variables analyzed were found in a previous study by López-de-Celis et al. [56] after a diacutaneous fibrolysis intervention. As previously mentioned, a minimum degree of muscular stiffness is necessary to ensure the transmission of muscle forces to the tendon. Reducing stiffness may not be the best way to improve athletic performance, at least in high-speed sports. Paradoxically, instead of activation, the pre-competition massage would be causing a “relaxing” effect, by increasing contraction time and decreasing stiffness and muscle tone.

Considering the transient duration of the effects of pre-competition massage on neuromuscular properties, it seems that it could have a greater influence in high-speed and short-duration sports. However, in endurance sports, the alteration of neuromuscular properties might be less relevant in terms of performance improvement. In this sense, pre-competition massage could have other objectives, such as improving blood flow perfusion [14].

As a limitation, it is important to highlight significant changes observed in the Tc and stiffness values observed also in the contralateral side (control leg). These changes could suggest that there is a cross-transfer effect, meaning that the effects of pre-competition massage may not appear only at a local level. Similar cross-transfer results have been observed in different parameters when performing passive stretching [57] and strength training [58,59,60]. In this sense, it is essential to carry out future studies with an independent control group, and not to use the contralateral limb as a control.

Moreover, it must be considered that these results are applicable only for this pre-competition massage protocol. It is necessary to carry out further studies with other types of massage, in order to draw general conclusions from this technique.

## 5. Conclusions

The main objective of pre-competition massage should be the improvement of athletic performance and reduction of the injury risk. This would imply increasing muscle tone and reducing contraction time, in order to prepare the athlete for a competition situation. In this study, data shows an increase in Tc and maximal radial displacement (Dm) variables, as well as a reduction in stiffness and tone. More quality studies are needed to draw clear conclusions about the effects of pre-competition massage.

## Figures and Tables

**Table 1:** Sample Characteristics.

Variable	N (%)—Mean (SD)
Sex	10 women (33.3%); 20 men (66.7%)
Age (years)	23.87 (6.17)
Weight (kg)	69.23 (1.76)
Height (cm)	174 (9.00)

**Table 2:** Outcomes intragroup.

		Baseline	Post-Intervention	Difference		
	Variable	Mean (SD)	Mean (SD)	Mean (SD)	*p*-Value	Effect Size
Experimental Limb						
Tensiomyography	Td	21.16 (1.93)	21.64 (1.83)	0.48 (1.64)	0.119 ^a^	0.26
	Tc	27.01 (8.53)	33.23 (11.64)	6.21 (10.36)	0.002 ^b^ *	0.61
	Ts	232.24 (81.34)	210.21 (56.74)	−22.03 (82.97)	0.237 ^b^	0.31
	Tr	65.92 (59.38)	53.91 (20.86)	−12.01 (51.78)	0.304 ^b^	0.27
	Dm	3.96 (1.11)	4.26 (1.27)	0.29 (0.86)	0.102 ^b^	0.25
Myotonometry	Tone	15.13 (1.44)	14.69 (1.33)	−0.43 (0.55)	0.001 ^a^ *	0.32
	Stiffness	260.88 (27.44)	252.55 (28.43)	−8.33 (11.67)	0.002 ^b^ *	0.30
	Relaxation	20.93 (2.08)	21.48 (1.90)	0.56 (1.05)	0.007 ^a^ *	0.28
Control Limb						
Tensiomyography	Td	20.57 (1.72)	20.96 (1.81)	0.39 (1.27)	0.106 ^a^	0.22
	Tc	25.97 (8.93)	27.72 (10.31)	1.75 (8.59)	0.049 ^b^ *	0.18
	Ts	203.43 (45.61)	208.02 (31.40)	4.59 (40.82)	0.060 ^b^	0.12
	Tr	44.22 (17.56)	56.13 (30.88)	11.92 (23.68)	0.008 ^b^ *	0.47
	Dm	4.31 (1.80)	4.22 (1.91)	−0.09 (0.71)	0.213 ^b^	0.05
Myotonometry	Tone	15.10 (1.31)	15.03 (1.23)	−0.06 (0.53)	0.506 ^a^	0.06
	Stiffness	259.92 (25.75)	255.87 (24.76)	−4.05 (9.40)	0.043 ^a^ *	0.16
	Relaxation	20.89 (2.25)	21.08 (2.19)	0.19 (0.60)	0.090 ^a^	0.09

Abbreviations: SD: standard seviation; ^a^: Paired *t*-test; ^b^: Wilcoxon test; *: Statistically significant; Td: delay time; Tc: contraction time; Ts: sustained time; Tr: relaxation time; Dm: maximal radial displacement.

**Table 3:** Differences between Groups.

	Variable	Mean (SD)	*p*-Value	Effect Size
Tensiomyography	Td	0.09 (1.76)	0.098 ^b^	0.37
	Tc	4.46 (10.25)	0.038 ^b^ *	0.50
	Ts	−26.61 (85.87)	0.019 ^b^ *	0.05
	Tr	−23.93 (54.94)	0.117 ^b^	0.08
	Dm	0.38 (1.00)	0.673 ^b^	0.03
Myotonometry	Tone	−0.37 (0.80)	0.048 ^b^ *	0.27
	Stiffness	−4.28 (12.52)	0.123 ^a^	0.13
	Relaxation	0.36 (1.09)	0.101 ^a^	0.20

Abbreviations: SD: standard deviation; ^a^: Unpaired *t*-test; ^b^: U Mann–Whitney test; *: Statistically significant; Td: delay time; Tc: contraction time; Ts: sustained time; Tr: relaxation time; Dm: maximal radial displacement.
